# Developing tools for analyzing and viewing multiplexed images

**DOI:** 10.1016/j.patter.2022.100549

**Published:** 2022-07-08

**Authors:** Sandhya Prabhakaran, Chandler Gatenbee, Alexander R.A. Anderson

**Affiliations:** 1 Department of Integrated Mathematical Oncology, H. Lee Moffitt Cancer Center and Research Institute, Tampa, FL 33612, USA

## Abstract

Dr. Prabhakaran and Dr Gatenbee are research scientists in Anderson’s lab and have developed Mistic, a publicly available tool that simultaneously views multiplexed images and assists in gaining biological and clinical insights into patients’ data. They discuss the role of mathematical modeling in translational cancer research and clinical decision making and describe how mathematical modeling fits into the data science definition.

## Main text

### What would you like to share about your background (personal and/or professional)?

**Alexander R.A. Anderson:** As a mathematician by training, I’ve always loved the idea that mathematics can help us better understand biology. My lab at Moffitt brings together a diverse team of quantitative scientists all viewing cancer through the lens of evolution and ecology. We integrate mathematical and computational modeling approaches with experimental and clinical data to better understand cancer and translate this understanding into novel therapies. Working with clinical data, it was inevitable that we would end up analyzing histology. I’m a Scotsman in love with New England IPA and an avid collector for indie rock vinyl (especially the colored variety) and vintage science fiction.

**Sandhya Prabhakaran:** I am a computer scientist and applied statistician by training. During my PhD and postdoctoral training, I was intrigued by the trifecta of problems in analyzing high-throughput biological datasets: the cost of collecting such data, the sheer size of the search space, and the complex and poorly understood mechanisms in biology. Solving these problems would require the right data and the usage of data science tools to help shape the biological questions, which in turn help build meaningful mathematical models to understand the underlying biological mechanisms. When I am not coding, I enjoy spending time with my family. I also practice yoga and am an avid runner.

**Chandler Gatenbee:** I am an evolutionary biologist by training and was bitten by the mathematical modeling bug during my PhD, where I used agent based models to explore how infection by the JC virus may increase the risk of colorectal cancer. After that, I took a deeper dive into modeling by joining the Department of Integrated Mathematical Oncology at Moffitt Cancer Center, which was especially appealing due to their focus on understanding cancer through the lens of evolution and ecology. This is also where I learned to work with whole slide images, as well as the ecological tools to analyze them. Outside of this work, I’m also a big IPA and music geek, always on the hunt for new brews and sounds.

### Why do you think mathematical models are important in translational cancer research?

**ARAA:** Cancers are complex, dynamic, adaptive systems—complex because they consist of multiple cellular and microenvironmental components, dynamic because the components interact with each other through a complex network of interactions that change in space and time, and adaptive because critical elements of the network as well as the network itself can change and adapt to perturbations. Mathematical models are truly the only way to decipher this complexity and predict potential therapeutic strategies to exploit it. Once you accept the dynamic adaptive nature of cancer, there is no escape cancer ecology and evolution—making sense of an evolving cancer with a spatially diverse ecology requires both sophisticated measurement technologies and also models that can utilize these measurements. Just as we have developed Mistic^1^ to visualize t-SNE with actual patient samples, we have also developed a suite of other tools to generate models (HAL,^2^ GATTACA^3^), analyze data (NeoPredPipe, VALIS^4^), and visualize them (EvoFreq, IsoMatrix). More details on all of the tools can be found here.

### What drew you to this area of research? How has the research focus of your team evolved over the years?

**ARAA:** Translation has always been a focus of my work at Moffitt and was one of the main reasons I moved my lab there in the first place. I really wanted mathematical models to impact clinical decisions, and working directly with clinicians and clinical data was the most obvious way to make that happen. This research focus has evolved as we gained a deeper understanding of resistance mechanisms and the realization that maximum-tolerated dose drives the evolution of resistance, so smarter evolution-informed treatment strategies needed to be developed.

### What kind of atmosphere do you look to foster in your team? Is there anything you try to replicate or avoid from your own experiences or that you have learned over the years?

**ARAA:** The integrated mathematical oncology (IMO) department has integrated in its name for a reason: everything we do is about integration across disciplines, and that includes a diversity of data sources across scales. That team science collaborative perspective permeates IMO, and it is how I try to run the lab. Collaborations between postdocs or even in small teams has emerged as a driving force for innovative and importantly productive science. Knowing how to put together the right team is a skill that takes time to master, and it is something I continue to learn from.

### Which achievement/discovery in your career are you most proud of?

**ARAA:** I’m particularly proud of getting a purely theoretical paper in *Cell*^5^ and of course the phase plane of a mathematical model on the cover of *Cancer Research*.^6^ More broadly, I’m incredibly proud to lead and have helped create the first mathematics department at the heart of an NCI designated cancer center.

### How did this project you wrote about come to be?

**SP:** Our project involved computationally analyzing multiple multiplexed images of lung cancer. We were interested in understanding the underlying spatial patterns between tumor and immune cells and how the tumors were organized. This required us to compare multiple images simultaneously and we realized there was no software (free or commercial) enabling this. We therefore generated an in-house image t-SNE for our purpose. Motivated by the usefulness of the image t-SNE in our work, we decided to build this as a standalone software called Mistic^1^ that can be used by the wider research community for simultaneous viewing of multiple two-dimensional images.

### Was there a particular element (paper, collaboration, talk/conference, key experiment, idea, result) that motivated you to start/participate in this project?

**SP:** We were fortunate enough to present our conceptual idea of Mistic as a lightning talk at the Cancer+DataViz Microlab held by NIH/NCI in November 2020, and this exposed us to the many challenges and gaps still present in the field of multiplexed image analysis. This motivated us to develop Mistic and make it available to the research community as an open source software.

### Was there a particular result that surprised you, or did you have a eureka moment? How did you react?

**SP:** Yes, there were a few eureka moments. When we realized that the image t-SNE had the visual power to segregate images into two biologically meaningful categories (patients who progressed and those that did not, given treatment), we were enthusiastic to build such a tool that would benefit the wider research community. I was also very thrilled when I was able to render the very first image t-SNE of 100 FoVS from our NSCLC data on the GUI by a series of frontend-backend calls, given that GUI programming and using Bokeh was new for me.

### What’s next for the project?

**SP:** We will enhance Mistic to use biologically meaningful regions of interest (ROIs) from the multiplexed image to render the overall image t-SNE. We also have plans to augment Mistic with other visualization software and build a cross-platform viewer plugin to improve the adoption, usability, and functionality of Mistic in the biomedical research community.

### When did you start preparing the manuscript? When did you decide to submit to *Patterns*?

**SP:** We started preparing the manuscript in fall 2021. We had reached out to the editor while preparing the manuscript to gauge the journal’s interest in our work. We submitted in December 2021.

### Why did you decide to publish in *Patterns*?

**ARAA:** We developed Mistic to help us find a pattern in the immune ecology of patients that was predictive of their response to immunotherapy, and so when it came to choosing a journal to publish Mistic, *Patterns* made perfect sense.

**SP:***Patterns* publishes groundbreaking research in data science, after a rigorous yet quick peer-review process. *Patterns* also has a global audience of data and computer scientists ensuring broader and faster research dissemination.

**CG:** I like that *Patterns* is domain agnostic and that the publications will be read by a wide audience interested in data science.

### What is the definition of data science in your opinion? What is a data scientist? Do you self-identify as one?

**ARAA:** I certainly identify as a mathematical oncologist or more generally a mathematical modeler. Data are certainly central for mathematical model calibration and validation, but mathematical models can be developed to generate hypothesis based on an understanding of the biological processes even without data. Data science to me is about analyzing data to gain scientific insights about a specific process or problem. In that sense, almost all scientists are data scientists!

**SP:** Data science is the skilled art of finding patterns in unstructured data. Anyone who can extract patterns by combining mathematics, statistics, and computer programming with domain expertise qualifies as a data scientist. I definitely identify as a data scientist.

**CG:** To me, data science is a fairly broad term and encompasses any approach to make discoveries with large datasets, including statistics, machine learning, computer vision, high-performance computing, integration into mathematical models, etc. I suppose I’ve never thought of myself as a data scientist, but I do use those approaches in my research, so maybe I should consider myself one.

### What motivated you to become a (data) researcher? Is there anyone/anything that helped guide you on your path?

**ARAA:** Data are integral to any of the translational work that we do. Mathematical models are only as good as the data that drive them. However, it wasn’t always like this; back in the 90s there wasn’t dialog between experimentalists and theoreticians and even less with clinicians. As our work has become more patient focused, we have integrated more and more patient-specific data, including clinical imaging, biopsies, and molecular biomarkers.

**SP:** I enjoy the challenge analyzing high-dimensional data brings and the learning opportunity it comes with. Data are very powerful if used in the right way. Data-driven decisions are becoming more and more commonplace. Data are also interdisciplinary, and it is an important yet rare skill to understand data in the context of these intersecting disciplines.

**CG:** I would say that I became a data researcher without even realizing it. When we’re developing our mathematical models, we often need to work with data, in order to generate and/or test hypotheses, calibrate, etc. Since my background is in evolution and ecology, I tend to think about inter- and intra-species interactions, something that can be studied in cancer through image analysis. In order to get and analyze the data to create and validate the models, I had to learn how to work with large datasets of whole slide images, which has been challenging but also very rewarding.

### How can data science help your domain?

**CG:** I think data science is critical in mathematical oncology. Being able to work with data enables one to generate hypotheses from the results of a data analysis, and/or test predictions from a mathematical model. It’s much easier to put faith into a model if there are some data to back up its assumptions and/or predictions.

### Which of the current trends in data science seem most interesting to you? In your opinion, what are the most pressing questions for the data science community?

**ARAA:** Single-cell genomics has been a major driver and will no doubt continue to be for some time, but I’m more excited about spatial transcriptomics as this has potential to allow for the understanding of interactions between cells and crucially gives them context. One of the big questions I see is how do we mechanistically bridge the diverse scales of the data we can currently collect? The genotype-phenotype map is the holy grail, but I’ll settle for one specific phenotypic trait and the mutations, proteins, and pathways that modulate it.

### How do you keep up to date with advances in both data science techniques and in your field/domain?

**SP:** Reading papers (suggested by Google scholar alerts, Twitter, podcasts), reviewing papers, attending conferences, participating and organizing workshops, hackathons, and special-interest working groups.

**ARAA:** In our field of mathematical oncology, we have specifically made a focused effort to develop community tools to share all of the current publications, conferences, and jobs through our weekly newsletter, blog, and YouTube channel.

### What is the fun part of being a data scientist?

**SP:** Being able to build models out of data that will ultimately be used for their predictive capabilities is incredibly exciting in my view. This predictive capability is the core of data science and paves the way to discovery and innovation. We tend to gain new insights or reinforce our knowledge while working with data.

**CG:** My favorite part of working with data, in my case images, is developing methods to extract something new from the data. It usually involves a lot of failures, so it’s exciting and rewarding when it finally works.

### What attributes make a data scientist successful?

**SP:** One needs to be inquisitive, creative, and patient to tame unstructured information and gather insight. Technical acumen and flexibility and statistical thinking are also key to make connections between different concepts and abstractions.

**CG:** Working with data, especially large datasets, can be extremely frustrating at times. So, I’d say an important attribute is patience. But, I think part of what can enable that patience is being passionate about your work and enjoying the process of discovery.

**Figure:**
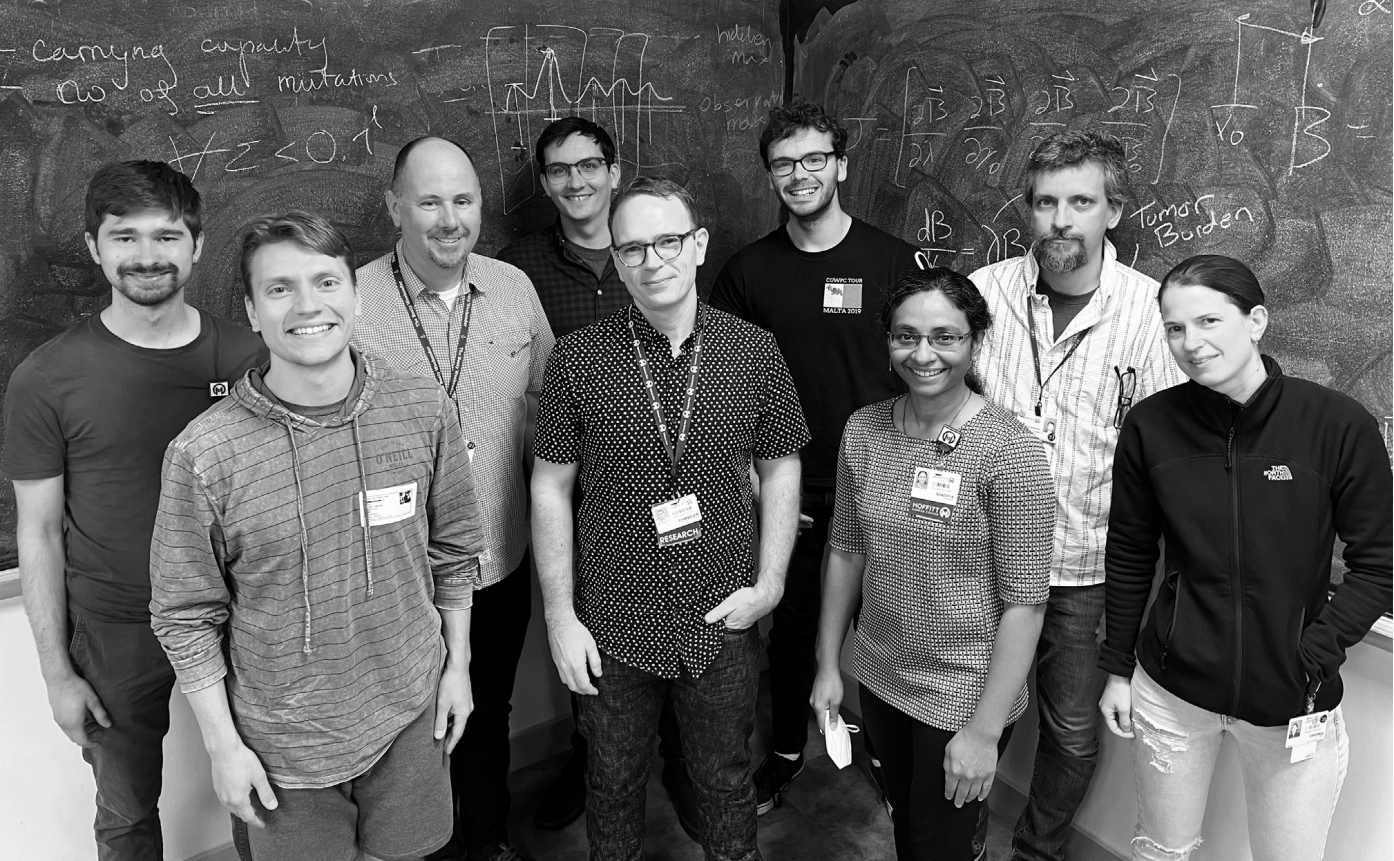
Subset of the Anderson Lab (from left to right): Maximilian Strobl, Luke Pierik, Sandy Anderson, Jeffrey West, Chandler Gatenbee, Kit Gallagher, Sandhya Prabhakaran, Mark Robertson-Tessi, and Virginia Turati.
